# Stimulatory effects of amino acids on γ-polyglutamic acid production by *Bacillus subtilis*

**DOI:** 10.1038/s41598-018-36439-4

**Published:** 2018-12-18

**Authors:** Chao Zhang, Daoji Wu, Xueliang Qiu

**Affiliations:** 1 School of Municipal and Environmental Engineering, Shandong Jianzhu University, Jinan, 250101 China; 2 Co-Innovation Center of Green Building, Jinan, 250101 China; 3 Shandong Futaste Co., Ltd, Yucheng, 251222 China

## Abstract

This paper is about study to increase the γ-PGA yield by developing new methods. The effect of various amino acids on production of γ-PGA by *Bacillus subtilis* Z15 was investigated. The γ-PGA yield was increased 23.18%, 12.15% and 31.46%, respectively, with 3 g/L aspartic acid (0 h), 1.5 g/L phenylalanine (0 h) and 7 g/L glutamic acid (24 h). Additonally, crude extract of glutamic acid after isoelectric crystallization (CEGA)could be a replacement for glutamate for γ-PGA production. Then, response surface methodology (RSM) was used for further optimization. The final media ingredient of amino acids were obtained as follow: CEGA 9 g/L, aspartic acid 4 g/L, phenylalanine 1.55 g/L. By applying this receipt in 5-L bioreactor, the γ-PGA yield reached 42.92 ± 0.23 g/L after 44 h, which is 63.1% higher than the control without amino acids for production. In addition, amino acids could shorten the lag phase and the average fermentation time (44 h versus 48 h). Fermentation with amino acids addition can be an positive option for γ-PGA production.

## Introduction

The γ-polyglutamic acid is a natural polymer composed of D-and L- glutamic acid units connected by γ-amide linkages^1^. This unusual anionic polymer is water soluble, water-absorbent, metal binding, biodegradable, edible and non-toxic to both human and environments^2–4^. These unique properties make γ-PGA have many broad application prospects in the fields of food, pharmaceutical, cosmetic, textile industries and agriculture^5–7^. However, the high cost of γ-PGA production is a major barrier to its commercialization, which is mainly associated with the low yield of γ-PGA. Aiming to the increasing applications of γ-PGA, it is necessary to increase the γ-PGA yield by developing new strategies^8–11^.

Amino acid is an important growth limiting factor, which can promote the growth of microorganisms and the synthesis of metabolic products. It has been widely used in the production process of many biochemical products such as biochemical drugs, enzyme preparations, antibiotics and so on. For example, Cruz *et al*.^12^ reported that high biomass production and high ethanol production were observed with the medium supplemented with 1% tyrosine for *Saccharomyces cerevisiae* A3. Wen *et al*.^13^ reported that the addition of cysteine, glutamic acid, glycine and serine showed a significant effect on glutathione accumulation of *S. cerevisiae* T65. By reference these papers, the suitable amino acids adding strategy was two-step: firstly, 2 mM cysteine was added after 2 h culture, and then the three amino acids (serine, glycine and glutamic acid) were added after 7 h culture. Using these amino acids addition strategy, glutathione yield reached 1.875 g/L, which was about 2.67 times more than that of no amino acids added.

The researches on the production of γ-PGA by microbial fermentation have been carried out for many years^14–16^. Some developed countries, such as the United States, Japan and South Korea, have made great progress in the preparation and application of γ-PGA, and many related processes and technologies have been patented. However, the effect of amino acids on the accumulation of γ-PGA by the Gram-positive model bacterium *B. subtilis* has not yet been reported^17,18^.

PGA-producing bacteria are divided into two types: L-glutamic acid dependent bacteria and L-glutamic acid independent bacteria. At present, most studies about γ-PGA production were focused on L-glutamic acid-dependent strain of *B. subtilis*. because of high yield^19,20^. We therefore chose *B. subtilis* Z15 for γ-PGA production in this study.

In this work, we tested the effect of various amino acids on γ-PGA production by *B. subtilis* Z15, and developed γ-PGA production through optimization of various of amino acids addition.

## Results

### Effects of single amino acid addition on biomass and γ-PGA production

The effects of various amino acids on γ-PGA production were listed in Table 1. According to the γ-PGA increase rate, amino acids was divided into the following four categories: (1) aspartic acid and phenylalanine could significantly improve the yield of γ-PGA (10%); (2) glutamic acid could promote the synthesis of γ-PGA (5~10%); (3) Additional of serine, arginine, proline, tyrosine, asparagine, isoleucine, histidine, arginine and valine had no obvious effect on γ-PGA synthesis (0~5%); (4) Additional of glutamine and lysine reduced the γ-PGA yield and biomass. Furthermore, with the increase of aspartic acid, phenylalanine and glutamic acid, the γ-PGA yield was increased 5.96%, 2.28% and 6.46% respectively. When the amounts of arginine and valine were increased, the γ-PGA yield showed no obviously change. So aspartic acid, phenylalanine and glutamic acid were chosen for the further research.

**Table 1:** Effect of amino acids on biomass and γ-PGA production.

Amino acids	Amount (g/L)	Biomass (g/L)	γ-PGA (g/L)	γ-PGAincrease rate (%)
Control	0	8.99 ± 0.24	26.32 ± 0.25	
Glycine	1	9.79 ± 0.21	27.10 ± 0.21	2.96
	2	10.25 ± 0.22	27.20 ± 0.18	3.34
Serine	1	8.81 ± 0.22	26.95 ± 0.12	2.39
	2	8.99 ± 0.20	27.01 ± 0.10	2.62
Glutamate	1	9.15 ± 0.33	28.32 ± 0.25	7.60
	2	11.36 ± 0.31	30.02 ± 0.21	14.06
Glutamine	1	8.45 ± 0.29	22.72 ± 0.25	−13.68
	2	8.13 ± 0.29	21.21 ± 0.15	−19.41
Histidine	1	8.51 ± 0.21	26.42 ± 0.25	0.38
	2	8.34 ± 0.20	26.23 ± 0.20	−0.34
Proline	1	8.41 ± 0.21	26.62 ± 0.25	1.14
	2	8.49 ± 0.11	26.78 ± 0.22	1.75
Tyrosine	1	8.65 ± 0.12	26.52 ± 0.25	0.76
	2	8.38 ± 0.19	26.28 ± 0.29	−0.15
Lysine	1	8.74 ± 0.22	25.92 ± 0.25	−1.52
	2	8.47 ± 0.18	25.74 ± 0.21	−2.20
Aspartic acid	1	9.75 ± 0.20	29.52 ± 0.25	12.16
	2	12.03 ± 0.29	31.09 ± 0.20	18.12
Asparagine	1	8.51 ± 0.21	26.42 ± 0.20	0.38
	2	8.78 ± 0.20	26.39 ± 0.20	0.26
Isoleucine	1	8.51 ± 0.21	26.22 ± 0.25	−0.38
	2	8.48 ± 0.18	26.37 ± 0.18	0.19
Phenylalanine	1	9.71 ± 0.29	28.85 ± 0.25	9.61
	2	11.70 ± 0.29	29.45 ± 0.20	11.89
Arginine	1	9.83 ± 0.22	27.22 ± 0.25	3.42
	2	9.77 ± 0.21	27.02 ± 0.21	2.66
Valine	1	9.69 ± 0.31	27.62 ± 0.25	4.94
	2	10.19 ± 0.23	27.52 ± 0.20	4.56

*Control group: *B. subtilis* Z15 is cultivated in the medium without amino acid addition.

As mentioned before, we chose a L-glutamic acid-dependent *B. subtilis* Z15 for γ-PGA production in this study due to high yield^19,20^. For glutamic acid-dependent bacteria, L-glutamic acid plays animportant role in the γ-PGA producing strains. The reason which affect the synthesis of γ-PGA with glutamic acid addition might be due to the insufficient amount of the precursor in the culture medium.

**Figure 1:**
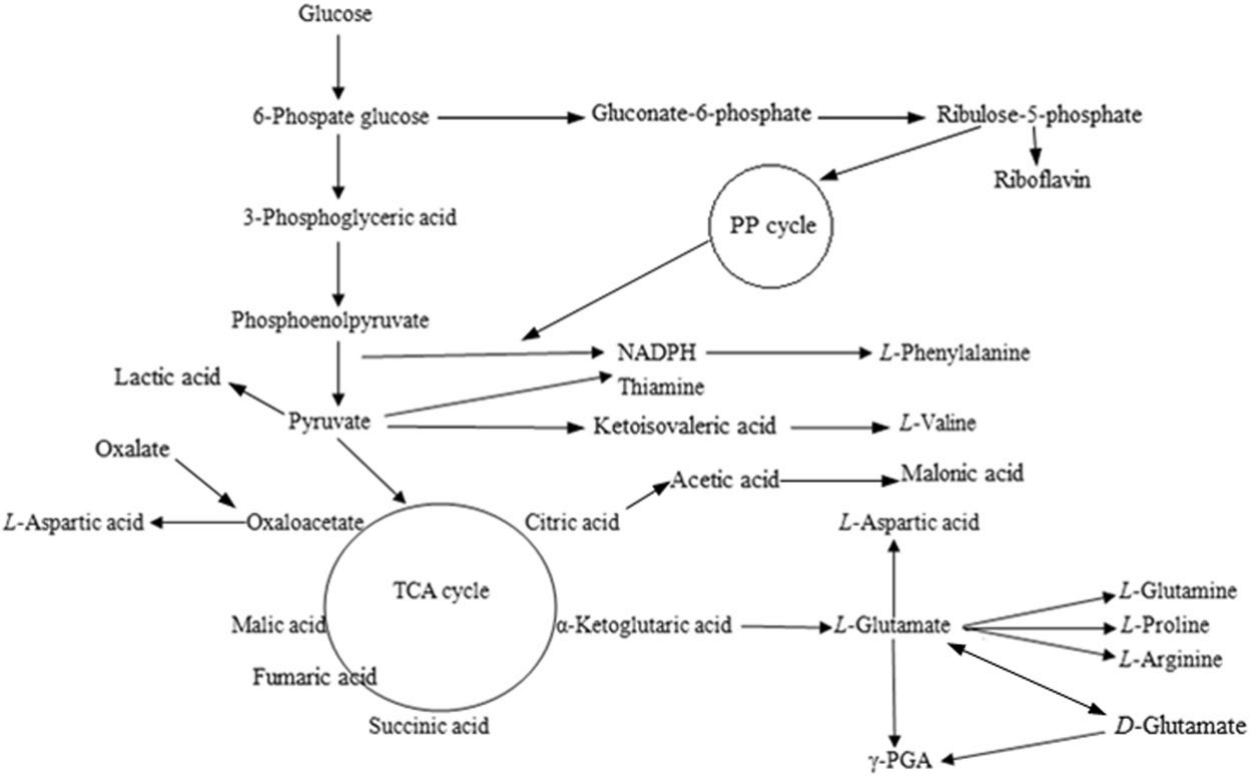
Metabolic network diagram of *B. subtilis*.

The metabolic pathways of γ-PGA are not fully clarified, and the probable metabolic pathways are shown in Fig. 1^21–26^. The synthesis of other amino acids is accompanied by the synthesis of γ-PGA. During the metabolic process of γ-PGA, L-glutamic acid is generated from α-ketoglutaric acid through three carboxylic acid (TCA) cycle. And part of L-glutamic acids is converted into D-glutamic acids. γ-PGA are synthesized by L-glutamic acids, D-glutamic acids and exogenous precursors. Addition of phenylalanine makes the metabolic flux to pyruvate. Therefore, more pyruvate enters the TCA cycle and do not inhibit the synthesis of pyruvate. Aspartic acid makes more oxaloacetate cycle in TCA. At the same time, the aspartic acid in synthesis pathway of γ-PGA requires a large amount of ATP, and the synthesis of ATP requires the participation of aspartic acid. The 2.0 g/L aspartic acid does not completely block the synthesis of oxaloacetic acid, aspartic acid, and ensure that the more amount of oxaloacetate in TCA cycle. In all, the addition of aspartic acid not only impact the block the synthesis of L-aspartic acid from oxaloacetic acid, but also ensures that oxaloacetic acid has more participation in TCA cycle.

### Effect of adding time of glutamic acid, aspartic acid and phenylalanine on γ-PGA production

**Figure 2:**
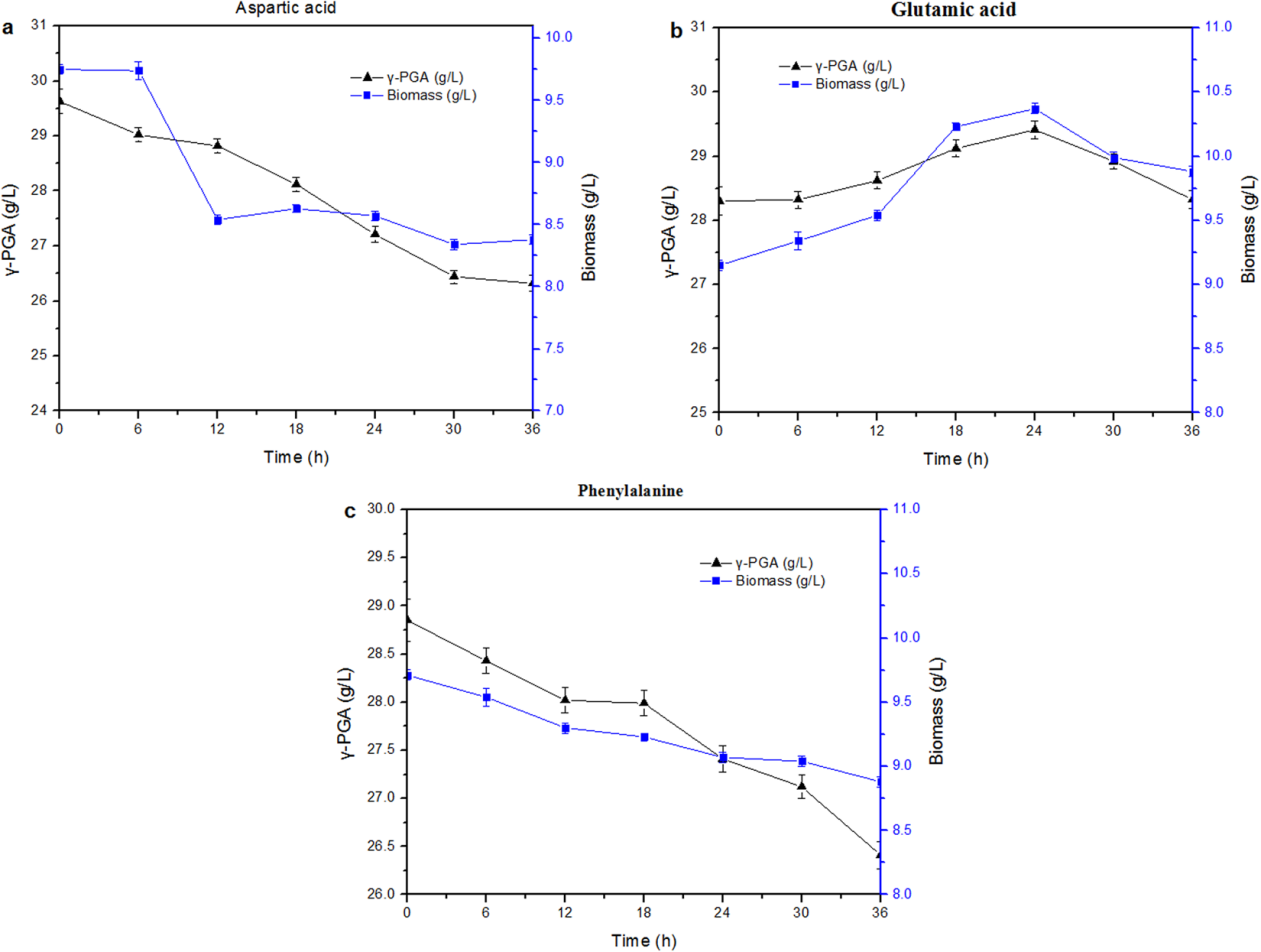
Effect of adding time of aspartic acid (**a**), glutamic acid (**b**) and phenylalanine (**c**) on γ-PGA production.

According to Table 1, further studies on glutamic acid, aspartic acid and phenylalanine which greater influence on the synthesis of γ-PGA were carried out. The influence of the adding time on the synthesis of γ-PGA was investigated (Fig. 1). In the *B. subtilis* Z15 broth, 0–10 h was lag phase, 10–48 h was exponential phase and 48–56 h was stationary phase(data not presented). So the addition time of amino acids were set as follows: 0 h, 6 h, 12 h, 18 h, 24 h, 30 h and 36 h. The concentrations of amino acids were 1 g/L. As shown in Fig. 2, the addition time of aspartic acid and phenylalanine was 0 h, and adding glutamic acid in the exponential phase (24 h) could promote the synthesis of γ-PGA.

In the process of γ-PGA synthesis, there is no need for a large amount of glutamic acid in the early stage of γ-PGA synthesis, as shown in Fig. 1. The early addition of glutamic acid may inhibit the synthesis of glutamic acid, or glutamic acid is used as a nitrogen source. Therefore, addition of glutamic acid in exponential phase is beneficial to the synthesis of γ-PGA. The results of Fig. 2 also prove the conjecture.

### Effect of concentration of amino acids on γ-PGA production

**Figure 3:**
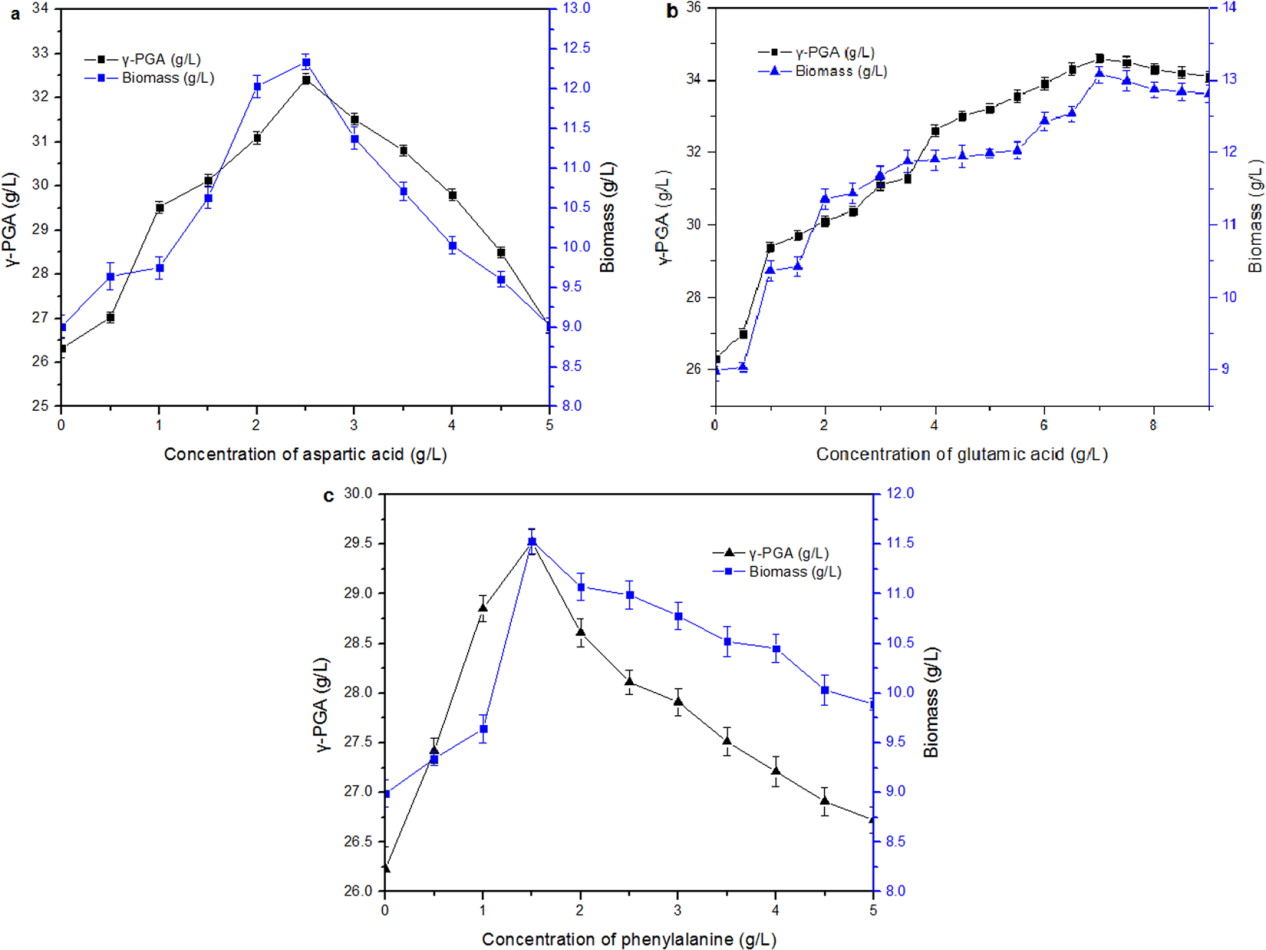
Effect of concentration of aspartic acid (**a**), glutamic acid (**b**) and phenylalanine (**c**) on γ-PGA production.

The effects of different concentrations of amino acids on the yield of γ-PGA were investigated. The results are shown in Fig. 3. Over more or over less acids are not impact to the accumulation of γ-PGA. The optimum concentrations of glutamic acid, aspartic acid and phenylalanine were 7 g/L, 3 g/L and 1.5 g/L, respectively.

### Effect of glutamic acid from different sources on γ-PGA production

As an important precursor of γ-PGA, large amount of glutamic acid should be added in the medium. In addition, 7 g/L glutamic acid still needed to be added at 24 h. So the whole fermentation process requires a large amount of glutamic acid. Whereas, it charges 50% of medium costs and was not economical for commercial scale production. Therefore, the precursors with low price were used studied to instead of refined monosodium glutamate.

Glutamic acid fermentation broth (GCFB), crude extract of glutamic acidafter isoelectric crystallization (CEGA) and crude monosodium glutamate after crystallization (CMG) were evaluated by the experiment. These amount of precursor substitutions were converted equally by the control group (L-glutamate, 60 g/L).

As shown in Table 2, CEGA, CMGand control group (t-test, data not presented) had the same beneficial effect on γ-PGA production, which suggests that CEGA and CMG were the ideal alternatives to glutamate. However, the price of CMG was a few times expensive compare with CEGA. So CEGA was chosen in the following experiments as a replacement for glutamate for γ-PGA production. GCFB had a negative effect on γ-PGA production, which may be impacted by some inhibitors in the fermentation broth.

**Table 2:** Effect of glutamic acid from different sources on γ-PGA production.

Precursors	γ-PGA (g/L)	Biomass (g/L)
Glutamic acid fermentation broth	28.98 ± 0.38	9.25 ± 0.23
Crude extract of glutamic acidafter isoelectric crystallization	34.42 ± 0.32	12.42 ± 0.33
Crude monosodium glutamate after crystallization	34.25 ± 0.31	12.21 ± 0.28
Control	34.60 ± 0.32	12.51 ± 0.30

### Box-Behnken design and response surface analysis

The optimal levels of the three amino acids (aspartic acid, phenylalanine and CEGA) were determined by Box-Behnken design (BBD). Levels of factors used in the Box-Behnken design are shown in Table 3, and the results are shown in Table 4. Variance for the quadratic design was analyzed to check the validity of the model (Table 5).

**Table 3:** Levels of factors used in the Box-Behnken design.

Factors	Level (g/L)		
	−1	0	1
(A) Aspartic acid	2	3	4
(B) Phenylalanine	1	1.5	2
(C) CEGA	5	7	9

**Table 4:** BBD experiments design matrix and results of γ-PGA production.

Code	Aspartic acid (A)	Phenylalanine (B)	CEGA (C)	γ-PGA (g/L)
1	−1	1	0	32.74 ± 0.20
2	1	1	0	39.26 ± 0.25
3	−1	0	−1	28.71 ± 0.23
4	0	1	1	37.09 ± 0.20
5	0	1	−1	32.23 ± 0.22
6	1	−1	0	38.56 ± 0.33
7	1	0	−1	36.92 ± 0.35
8	0	0	0	35.75 ± 0.15
9	0	−1	−1	29.59 ± 0.33
10	−1	−1	0	28.07 ± 0.10
11	0	0	0	36.86 ± 0.33
12	0	0	0	36.63 ± 0.14
13	0	0	0	36.27 ± 0.15
14	0	0	0	36.04 ± 0.13
15	−1	0	1	36.10 ± 0.17
16	1	0	1	42.88 ± 0.30
17	0	−1	1	34.11 ± 0.25

**Table 5:** ANOVA of RSM.

Source	Sum of Squares	Mean Square	F Value	Probe (P) > F
Model	238.24	26.47	56.48	<0.0001
A	128.00	128.00	273.09	<0.0001
B	15.10	15.10	32.21	0.0008
C	64.58	64.58	137.79	<0.0001
AB	3.94	3.94	8.41	0.0230
AC	0.51	0.51	1.09	0.3310
BC	0.029	0.029	0.062	0.8110
A^2^	1.63	1.63	3.48	0.1043
B^2^	21.79	21.79	46.49	0.0002
C^2^	2.56	2.56	5.47	0.0520
Residual	3.28	0.47	4.18	0.1003
Lack of Fit	2.49	0.83		
Pure Error	0.79	0.20		
Cor Total	241.52			

Values of “Prob > F” less than 0.0500 indicates model terms are significant. In this case A, B, C, BC, A^2^ are significant model terms. Final equation in terms of coded factors is stated in the following equation:

1 $$\begin{array}{rcl}{\rm{Y}} & = & 36.31+4\times {\rm{A}}+1.37\times {\rm{B}}+2.84\times {\rm{C}}-0.99\times {\rm{A}}\times {\rm{B}}-0.36\times {\rm{A}}\times {\rm{C}}\\  &  & +\,0.085\times {\rm{B}}\times {\rm{C}}+0.62\times {{\rm{A}}}^{2}-2.28\times {{\rm{B}}}^{2}-0.78\times {{\rm{C}}}^{2}\end{array}$$

where Y is γ-PGA yield, A is aspartic acid; B is phenylalanine, and C is CEGA.

This result clearly indicated that experimental values were distributed linearly with high correlation (R^2^ = 0.9864). Therefore, the model was adequate for prediction of γ-PGA value within the range of variables studied. The maximum γ-PGA yield of 42.66 g/L was obtained at the condition of 4 g/L aspartic acid, 1.55 g/L phenylalanine and 9 g/L CEGA. To validate this prediction,three independent fermentations were carried out, and a γ-PGA yield of 42.92 ± 0.27% was obtained.

**Figure 4:**
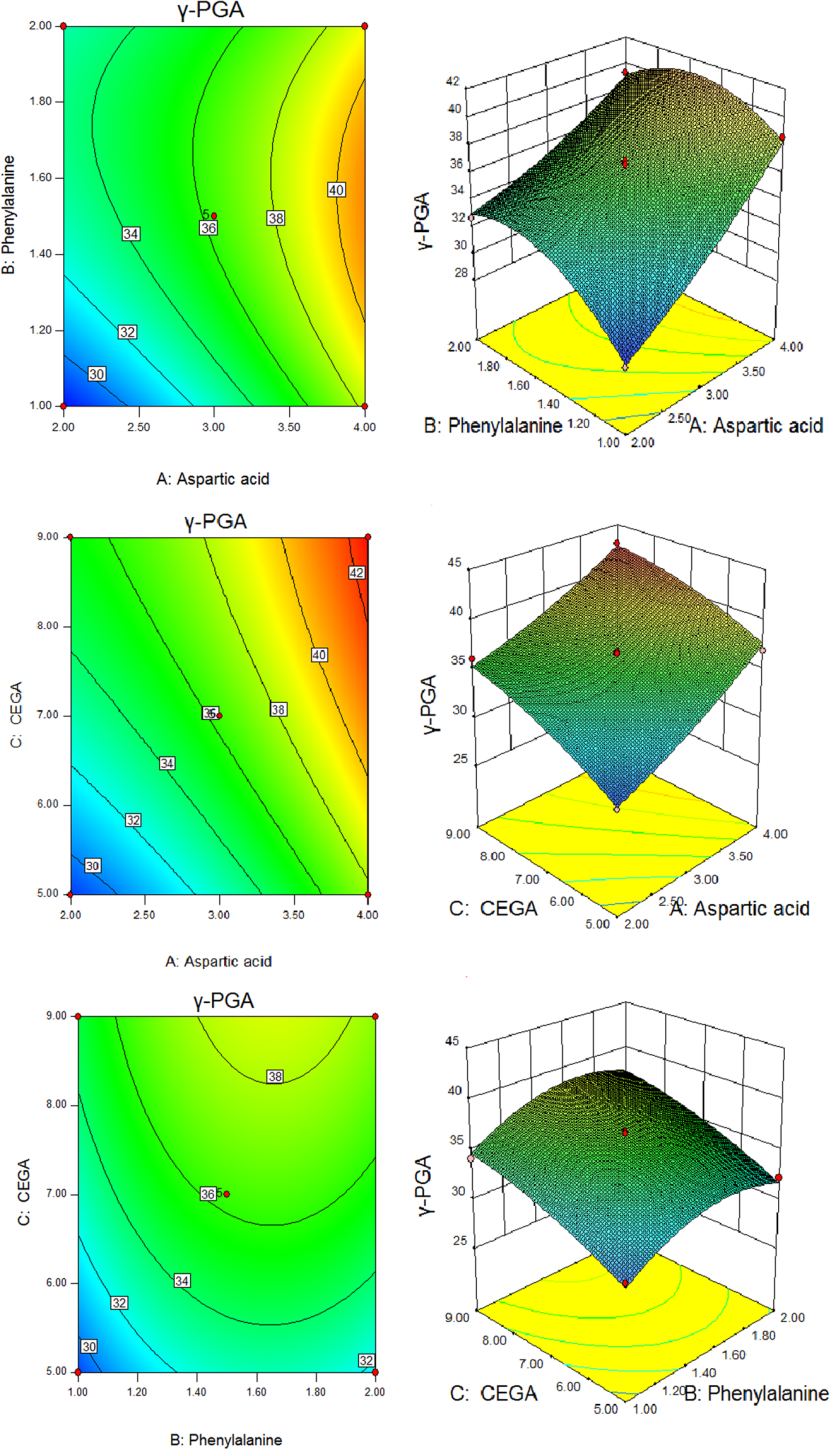
Surface and contour plots of mutual-influence. (1) Effect of aspartic acid (**A**) and phenylalanine (**B**); (2) effect of aspartic acid (**A**) and CEGA (**C**); (3) effect of phenylalanine (**B**) and CEGA (**C**).

The effects of the three variables (aspartic acid, phenylalanine and CEGA) on the production of γ-PGA were analyzed using RSM. 3D response surface plots and contour plots were studied to investigate the interaction effects of any two variables on the response (Fig. 4).

### Batch cultivation in 5-L bioreactor

**Figure 5:**
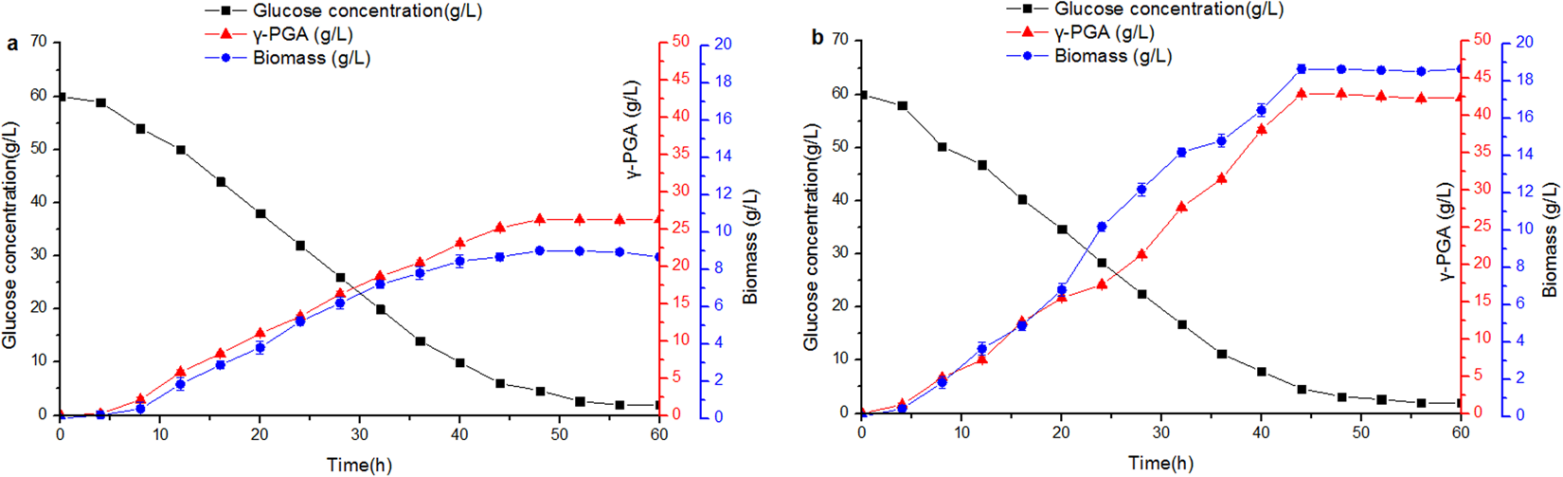
Batch cultivation in 5-L bioreactor.

According to the above results obtained inthe flask, the potential for utilizing the amino acids adding strategy for γ-PGA production were investigated in this section. Therefore, the batch fermentation with amino acids were carried out in 5-L bioreactor. Figure 5(b) presented the fermentation profiles (glucose consumption, cell growth and γ-PGA production) for *B. subtilis* Z15 with amino acid addition. For comparison, Fig. 5(a) presented the fermentation process of *B. subtilis* Z15 without amino acids addition. As shown in Fig. 5(a), with the additional supplement of amino acids intothe broth, *B. subtilis* Z15 growth in the broth was observed in 4 h, which is shown in 8 h in the broth of *B. subtilis* Z15 without amino acids addition. This indicated that the presence of amino acids can shorten the lag phase. After 4 h of cultivation, the cells started to grow, when the concentration of reducing sugar began to decrease. Meanwhile, γ-PGA was secreted to the broth, the concentration of γ-PGA rapidly increased during exponential phase and reached a plateau in the stationary phase. γ-PGA production was accompanied by the simultaneous consumption of reducing sugar. No further γ-PGA was produced after the depletion of reducing sugar. Final cell concentration was 18.67 ± 0.20 g/L and γ-PGA production was 42.92 ± 0.23 g/L after 44 h. The results showed that γ-PGA produced by *B. subtilis* Z15 was associated partially with cell growth. The average fermentation time was decreased from 48 h to 44 h with amino acids addition, with less residual sugars in the culture at the end of fermentation. Considering all these results, the amino acids addition strategy can be significant improve process for γ-PGA production. Due to aspartic acid and phenylalanine are used less, and there are good alternatives to replace large amounts of glutamic acid, the cost of the culture medium can be significantly reduced.

## Discussion

The purpose of this study was to add various amino acids on the production of γ-PGA by *B. subtilis* Z15, and to analyze its mechanism. However, there are different mechanisms of action after adding amino acids in the production of many fermented products, which can be classified into four types. The first type, the carbon skeleton formed amino acid after deamination enters the TCA cycle through a specific metabolic pathway, increasing the metabolic flux of the TCA cycle, thus improving the cell’s energy level, maintaining cell growth or metabolites to synthesize^2,3^. The second types, because amino acid is a zwitterion, it has the function of regulating intracellular pH^13,14^. The third type is inhibiting or relieving the activities of key enzymes in related metabolic pathways, thereby promoting the production of target metabolites^1,15,16^. The fourth type, amino acids can produce coenzyme or intermediate metabolites (including precursor) through specific metabolic pathways^4,17^, which is needed in the target metabolic pathway. As can be seen from Fig. 1^21–26^, the synthesis of other amino acids is accompanied by the synthesis of γ-PGA. During the metabolic process of γ-PGA, L-glutamic acid is generated from α-ketoglutaric acid through TCA cycle. And part of L-glutamic acids are converted into D-glutamic acids. γ-PGA are synthesized by L-glutamic acids, D-glutamic acids and exogenous precursors. Addition of phenylalanine makes the metabolic flux to pyruvate. Therefore, more pyruvate enter the TCA cycle and do not inhibit the synthesis of pyruvate. Aspartic acid may feed into the TCA cycle, thereby increasing flux towards oxaloacetate cycle. At the same time, the aspartic acid in synthesis pathway of γ-PGA requires a large amount of ATP, and the synthesis of ATP requires the participation of aspartic acid. The 2.0 g/L aspartic acid does not completely block the synthesis of oxaloacetic acid, aspartic acid, and ensure that the more amount of oxaloacetate in TCA cycle. In summary, amino acids could significantly improve γ-PGA production in the process of *B. subtilis* fermentation, and its mechanism of action should belong to the fourth type.

In this study, the effect of various amino acids on production of γ-polyglutamic acid by *B. subtilis* Z15 was investigated. The γ-PGA yield was increased 23.18%, 12.15% and 31.46%, respectively, with 3 g/L aspartic acid (0 h), 1.5 g/L phenylalanine (0 h) and 7 g/L glutamic acid (24 h). In addition, CEGA had the same beneficial effect on γ-PGA production, which suggests that CEGA was ideal alternatives to glutamate. Then, RSM was adopted for further optimization. The concentrations of amino acids were obtained as follow: CEGA 9 g/L, aspartic acid 4 g/L, phenylalanine 1.55 g/L. By applying this amino acid addition strategy further to batch fermentation in 5-L bioreactor, the γ-PGA productionreached 42.92 ± 0.23 g/L after 44 h, which is 63.1% higher than the control without amino acids addition. Furthermore, the addition of amino acids could shorten the lag phase and the fermentation time (44 h versus 48 h).

Precursors (Intermediates in the PGA biosynthesis), PGA yield and special remarks for PGA-producing strains is summarized in Table 6. Citric acid is considered as the best precursor for the production of PGA^27–30^. Bajaj and Singhal screened various TCA cycle intermediates likeα-ketoglutaric acid, malic acid, succinic acid or pyruvic acid for production PGA by *B. licheniformis* NCIM 2324 and found the yield of PGA to increase from 26.12 to 34.98 g/l after addition of 10 mM ofα-ketoglutaric acid in the production medium. Bajaj and Singhal found 0.5 mM L-glutamine to support maximum PGA production. The yield of PGA further increased with combined addition of glutamine and α-ketoglutaric acid to the medium^30^. However, the increase of PGA output in the above study is not as good as that in this study. Although this technology requires exogenous addition of amino acids (aspartic acid, phenylalanine and glutamic acid), the addition of aspartic acid and phenylalanine is used less, and there are good alternatives to replace large amounts of glutamic acid, the cost of the culture medium can be significantly reduced. Therefore, fermentation with amino acids addition can be a cost-effective option for γ-PGA production.

**Table 6:** Effect of different metabolic precursors on the production of γ-PGA.

Strain	Metabolic precursor	γ-PGA (g/L)	Remarks	Reference
*B. licheniformis* ATCC 9945	Glutamic acid, citric acid	23.00	Utilized citrate rapidly at pH 6.5 which translated in to increased PGA production.	^27^
*B. subtilis* R 23	Glutamic acid, citric acid (Fed batch)	35.00	Pulsed-feeding of citric acid (1.44 g/L/h) and L-glutamic acid (2.4 g/L/h) when citric acid was depleted.	^28^
	Glutamic acid, citric acid, α-ketoglutaric acid	25.38	Addition of α-ketoglutaric acid to the medium increased yield as well as molecular of PGA.	^29^
*B. licheniformis* NCIM 2324	Glutamic acid, citric acid, α-ketoglutaric acid, glutamine	35.75	Addition of α-ketoglutaric acid and glutamine to the medium increased yield as well as molecular of PGA.	^30^
*B. subtilis* Z15	CEGA, aspartic acid, phenylalanine	42.92	Crude extract of glutamic acid after isoelectric crystallization (CEGA) could be a replacement for glutamate for γ-PGA production.	This study

## Materials and Methods

### Strain

*B. subtilis*Z15 (CICC 20643) was obtained from China Center of Industrial Culture Collection.

### Semi-finished products of monosodium glutamate industry

The glutamic acid fermentation broth (glutamic acid, 8.04 ± 0.15 g/L), crude extract of glutamic acidafter isoelectric crystallization (glutamate mass fraction, 80.09 ± 0.24%) and crude monosodium glutamate after crystallization (glutamate mass fraction, 90.16 ± 0.19%) were collected from Bioengineering Experiment Center of Shandong Jianzhu University, Jinan, China, which were stored at 4 °C.

### Media

Slant medium (SM), in g/L: glucose, 20; peptone, 10; sodium glutamate, 20; NaCl, 5; agar 18.

Seed medium (SM), in g/L: glucose, 20; peptone, 10; sodium glutamate, 20; NaCl, 5. The pH was adjusted to 7.0.

The optimal fermentation medium without semi-finished product for *B. subtilis* Z15, in g/L: glucose, 60; peptone, 30; sodium glutamate, 60; NaCl, 15. The pH was adjusted to 7.0 by HCl or NaOH.

Fermentation medium (FM), in g/L: glucose, 60; peptone, 30; crude extract of glutamic acid, 75; NaCl, 15. The pH was adjustedto 7.0.

These media were autoclaved at 121 °C for 20 min.

### Shake flask experiment

A loop of bacterias from the slant were transferred into 250 mlflask containing 30 ml seed medium. Seedculture were incubated ona rotary shaker operating (200 r/min) for 24 hr at 37 °C.

*B. subtilis* Z15 was performed in a 250 ml flaskcontaining 50 ml fermentation medium inoculatedwith 10% (v/v) of the seed culture. Culture conditions of agitationrate, temperature and growth period were set at 200 r/min, 37 °C, and 48 h, respectively.

### BBD and response surface analysis

BBD was used to identify optimal concentrations of three amino acids using Design-Expert software(Version 8.0.6, Stat-Ease, Inc, USA). Three factors were aspartic acid (A), phenylalanine (B)and glutamicacid (C). Evaluated response Y is γ-PGA production (g/L). The three factors were studied at three different levels (Table 3) and sets of 20 experiments were carried out (Table 4).

### Batch cultivation in 5-L bioreactor

Batch cultivation was carried out in 5-L bioreactor (Baoxing Corp., Shanghai, China). The liquid filling quantity of bioreactor was 60% (v/v). The pH, agitation and temperature of the broth were maintained at 7.0, 200 r/min and 37 °C, respectively. The fermentation process lasted for 60 h. Samples were taken for biomass accumulation and γ-PGA production analysis every 4 h.

### Analytical methods

The yield of γ-PGA was measured by gel permeation chromatography (GPC) system following the method reported previously^31^. The glucose contents were estimated by the dinitrosalicylic acid method^32^.

Bacteria biomass was estimated by bacteria dry weight. The fermentation broth (30 ml) was centrifuged (12,000 × g) for 10 min. The bacteria were washed twice with distilled water and dried at 105 °C overnight.
